# Effect of maternal death on child survival in rural West Africa: 25 years of prospective surveillance data in The Gambia

**DOI:** 10.1371/journal.pone.0172286

**Published:** 2017-02-22

**Authors:** Susana Scott, Lindsay Kendall, Pierre Gomez, Stephen R. C. Howie, Syed M. A. Zaman, Samba Ceesay, Umberto D’Alessandro, Momodou Jasseh

**Affiliations:** 1 Medical Research Council, The Gambia Unit, Fajara, The Gambia; 2 London School of Hygiene and Tropical Medicine, London, United Kingdom; 3 Department of Paediatrics, University of Auckland, Auckland, New Zealand; 4 Centre for International Health, University of Otago, Dunedin, New Zealand; 5 Ministry of Health and Social Welfare, Banjul, The Gambia; Institut Pasteur, FRANCE

## Abstract

**Background:**

The death of a mother is a tragedy in itself but it can also have devastating effects for the survival of her children. We aim to explore the impact of a mother’s death on child survival in rural Gambia, West Africa.

**Methods:**

We used 25 years of prospective surveillance data from the Farafenni Health and Demographic surveillance system (FHDSS). Mortality rates per 1,000 child-years up to ten years of age were estimated and Kaplan-Meier survival curves plotted by maternal vital status. Cox proportional hazard models were used to examine factors associated with child survival.

**Findings:**

Between 1^st^ April 1989 and 31^st^ December 2014, a total of 2, 221 (7.8%) deaths occurred during 152,906 child-years of follow up. Overall mortality rate was 14.53 per 1,000 child-years (95% CI: 13.93–15.14). Amongst those whose mother died, the rate was 25.89 (95% CI: 17.99–37.25) compared to 14.44 (95% CI: 13.84–15.06) per 1,000 child-years for those whose mother did not die. Children were 4.66 (95% CI: 3.15–6.89) times more likely to die if their mother died compared to those with a surviving mother. Infants whose mothers died during delivery or shortly after were up to 7 times more likely to die within the first month of life compared to those whose mothers survived. Maternal vital status was significantly associated with the risk of dying within the first 2 years of life (p-value <0.05), while this was no longer observed for children over 2 years of age (P = 0.872). Other factors associated with an increased risk of dying were living in more rural areas, and birth spacing and year of birth.

**Conclusions:**

Mother’s survival is strongly associated with child survival. Our findings highlight the importance of the continuum of care for both the mother and child not only throughout pregnancy, and childbirth but beyond 6 weeks post-partum.

## Introduction

Since the initiation of the Millennium Development Goals (MDGs), there has been considerable progress in reducing maternal and child mortality. Between 1990 and 2010, global maternal mortality has decreased by nearly 50%[1, 2], and the average annual rate of reduction in child mortality has more than doubled in the past decade compared to the previous decade[3]. In spite of this, maternal mortality remains high in low-income countries, particularly in rural and less accessible areas. Many of the health interventions developed over the last decade have focused on urban areas, and rural areas have struggled to successfully scale up and sustain these interventions. The death of a mother is a tragedy in itself and it can have devastating effects for the survival of her children. A mother’s death, especially within the first few months of a child’s life, compromises nutrition (due to interruption of breastfeeding) and overall child care, and these children are less likely to attend routine health services and benefit from preventive interventions such as vaccination. This effect is exacerbated in poor and underserved communities.

Several studies in sub-Saharan African countries with high Human Immunodeficiency Virus (HIV) prevalence have shown the effect of an HIV infected mother’s death on the risk of her child dying[4–7]. In a pooled analysis of nine clinical trials, the adjusted odds of dying was 2.27 higher in children born to HIV-infected mothers who died compared those whose mothers were still alive[4]. However, few studies have looked at this issue in sub-Saharan African populations with low HIV prevalence[8, 9]. Studies of the long term impact of parental death on child survival are few and also conflicting. A study in Bangladesh found that the cumulative probability of survival to age 10 years was 24% in children whose mothers died, compared with 89% in those whose mothers remained alive[10]. In contrast, historical data between 1950 and 1974 from West Kiang, The Gambia, showed little effect after 2 years of age[9]. Since this historical paper in The Gambia, there have been many epidemiological changes in both disease and infection in this region largely as a result of improved health care programmes, including increased childhood vaccination coverage[11] and strengthened malaria control programmes[12]. Overall, child mortality has decreased but like many areas in sub-Saharan Africa, there has been less improvement in neonatal survival[12]. Maternal mortality has also remained very high in this region, at 461 per 100,000 live births[13]. Considering the greatest risk of death for a child is within the first month of life and the largest impact of a mother dying on child survival occurs in the same time period[4, 9, 10], it is intuitive that reducing maternal mortality would improve child survival.

The Farafenni Health and Demographic Surveillance System (FHDSS) is the one of the oldest demographic surveillance sites in sub-Saharan Africa, with over three decades of prospective surveillance[14]. The aim of this study is to use this valuable data resource for exploring the impact of a mother dying on child survival in rural areas in West Africa.

## Methods

### Study population and data collection

Gambia is a small West African country with a total population of under 2 million, a gross national income per capita of $1,620 and an under five mortality rate of 98 per 1000 live births (http://www.who.int/countries/gmb/en/). The FHDSS was established in 1981 and details have been previously published[14]. Briefly, the FHDSS covers a rural area of North Bank Region in The Gambia, which includes 42 rural villages, the small town of Farafenni and the area within a 5km radius of the town. The majority of residents are Muslims and subsistence farmers with few earning salaries from employment. The health care system consists of one regional hospital, one health centre, 16 primary health care posts and five dispensaries. Over the past 20 years, under-5 mortality has decreased, but with little improvement to neonatal mortality [12].

Residents live in compounds which are demarcated by a fence. Each compound is made up of a number of households. In the rural villages, most compounds have single households. However, in Farafenni town, it is common to see several households within each compound. A household is defined as a group of people living in the same house or compound, sharing the same cooking arrangements. The total population under surveillance as of December 2012 was 50 455 living in 6668 households: 17% of the residents are less than 5 years of age and just under 50% are less than 15 years of age. Data on births, deaths, and migration are collected routinely every 4 months from each household within the surveillance area. These include information on how individuals enter the surveillance population (through initial enumeration, birth in the area or in-migration), how they leave (death or outmigration), date of exit, gender and ethnicity. Each individual is given a unique identity number that does not change. At each three-four monthly round, each household is asked if any person has moved out and where. Once the new address is identified and if within the surveillance area, the DHSS field worker covering the new village confirms this and adds the new person to the relevant household, limiting any risk of double counting person-time.

### Statistical analysis

With the exception of a 13-month period between February 2008 and March 2009 surveillance has been uninterrupted since 1981. However, since 1989, fieldworkers were required to visit every compound under surveillance at least once every quarter[14]. Due to this change in data collection methods, which also improved the quality of the data, this analysis only includes births from 1^st^ April 1989 to 31^st^ December 2014. Each resident under surveillance has a unique identification number (comprising a village code, a compound number, a household number and a personal number). Where known, they are linked with their mother.

Mortality rates per 1,000 child-years were estimated and Kaplan-Meier survival curves plotted by maternal vital status (alive or dead). All children up to ten years of age were censored at time of death, at the date of their last successful follow-up visit or the date when their mother exited the surveillance area (after which point her vital status was unknown). The data were split at the time of a mother’s death and subsequent survival was assessed. Rate ratios (with 95% confidence intervals) and results from Cox proportional hazard models were used to compare childhood mortality by maternal vital status. To allow for multiple births, a mother’s identifying code (ID) was fitted as a clustering variable. After fitting an initial Cox proportional hazard model, and adjusting for clustering on mother ID, the impact of maternal vital status varied significantly by age group of the child (global proportional-hazards assumption test p<0.001). As a result we split survival time into the following 6 age groups, birth to <1 week (early neonatal period), 1 week to <1 month (late neonatal period), 1 month to <6 months, 6 months to <1 year, 1 year to <2 years and > = 2–10 years. Cox regression analysis was used to examine factors associated with child survival. These factors include gender, ethnic group, year of birth, mother’s age at birth, region, birth order and birth spacing to nearest sibling. Statistical analyses were performed using STATA 14.0 statistical software (StataCorp LP, USA, http://www.stata.com).

### Ethical approval

The Joint MRC/Gambia Government Ethics committee approved the establishment of the Farafenni HDSS and all instruments used to collect household and individuals level information. Consent was given at community level by the community leaders. However, each household has the right to refuse if they do not wish to participate in the surveys.

## Results

Between 1^st^ April 1989 and 31^st^ December 2014, a total of 29,641 births were recorded within the study area of which 29,238 (98.6%) had maternal vital status information available. Of these, 736 (2.5%) were excluded for failing data consistency checks, resulting in a final analysis dataset of 28,502 individuals from 3,567 compounds in 115 villages. Baseline characteristics, separated by child’s vital status, are detailed in Table 1. The median age (and duration) in the follow up period was 5.02 years (IQR: 2.09–9.23). Five thousand and thirty three children (17.7%) left the study area before the reaching 10 years of age (Table A in S1 File); their mean age was 4.02 years (SD: 2.7). Three thousand, five hundred and ninety-three (12.6%) mothers left the study area during the study period. Women who left before the study end were younger at time of their child’s birth (mean: 25.09 years, SD:6.4) compared to those who remained until their child was 10 years of age (mean 28.03 years, SD: 7.3), ttest, P<0.001.

**Table 1:** Baseline characteristics by child’s vital status.

Characteristics	Child alive		Child died	
	n	%	n	%
**Total**	26,281	92.2	2,221	7.8
**Mother vital status**				
** Alive**	25,930	98.7%	2,192	98.7%
** Dead**	351	1.3%	29	1.3%
**Sex**				
** Male**	13,380	50.9%	1,177	53.0%
** Female**	12,901	49.1%	1,044	47.0%
**Ethnic group**				
** Wolof**	11,367	43.3%	12,354	43.3%
** Mandinka**	8,302	31.6%	9,022	31.7%
** Fula**	5,687	21.6%	6,155	21.6%
** Other**	925	3.5%	971	3.4%
**Year of birth**				
** 1989–1995**	3,170	12.1%	714	32.1%
** 1996–2000**	2,525	9.6%	453	20.4%
** 2001–2005**	5,515	21.0%	432	19.5%
** 2006–2010**	7,983	30.4%	378	17.0%
** 2011–2014**	7,088	27.0%	244	11.0%
**Mother's age at birth**				
** <20 years**	3787	14.4%	353	15.9%
** 21–30**	13349	50.8%	947	42.6%
** 31–45**	8817	33.5%	866	39.0%
** 45+**	328	1.2%	55	2.5%
** mean (sd)**	27.66	7.27	28.5	8.49
**Region**				
** Rural**	14,908	56.7%	1,788	80.5%
** Urban**	11,373	43.3%	433	19.5%
**Birth order**				
** 1**	10,219	38.9%	872	39.3%
** 2**	6,193	23.6%	532	24.0%
** 3**	3,927	14.9%	332	14.9%
** 4**	2,501	9.5%	232	10.4%
** 5**	1,455	5.5%	127	5.7%
** 6+**	1,986	7.6%	126	5.7%
**Birth spacing with closest younger sibling**				
** <18 months**	1,334	5.1%	212	9.5%
** 18–36 months**	9,804	37.3%	743	33.5%
** >36 months**	4,924	18.7%	394	17.7%
** No younger sibling**	10,219	38.9%	872	39.3%
**Birth spacing with closest older sibling**				
** <18 months**	804	3.1%	376	16.9%
** 18–36 months**	9,813	37.3%	853	38.4%
** >36 months**	5,028	19.1%	374	16.8%
** No older sibling**	10,636	40.5%	618	27.8%

### Survival analysis

A total of 2,221 (7.8%) deaths occurred during 152,906 child-years of follow up. This equates to a rate of 14.53 per 1,000 child-years (95% CI 13.93–15.14). Splitting these deaths by maternal vital status gives 2,192 deaths in 151,785 child-years where the mother is alive, a rate of 14.44 per 1,000 child years (95% CI 13.84–15.06), and 29 deaths in 1120 child-years where the mother is dead, a rate of 25.89 per 1,000 child-years (95% CI 17.99–37.25). Overall, children were 4.66 times more likely to die if their mother died compared to those with a surviving mother (unadjusted HR: 4.66 95% CI: 3.15–6.89).

The overall median interval between death of the mother and death of the child was 91 days (IQR:17–303). Among the mothers who died before their children 44.8% (13/29) occurred at delivery, with their children dying on average 18 days later (IQR:3–58). For those mothers who died before their child but not at delivery, the median interval between mother and child dying was 243 (IQR:40–483) days. Among the women who died, those who died before their children’s’ death were younger (mean 30.2 years, SD 7.7) compared to those who were survived by their children (mean 36.7, SD 9.8, t-test, p = 0.0005). The proportion of women aged less than 20 years was significantly higher in mothers who died before their children (16.3%) compared to those who died but were survived by their children (1.5%) (χ2 test, p<0.0001).

**Fig 1:**
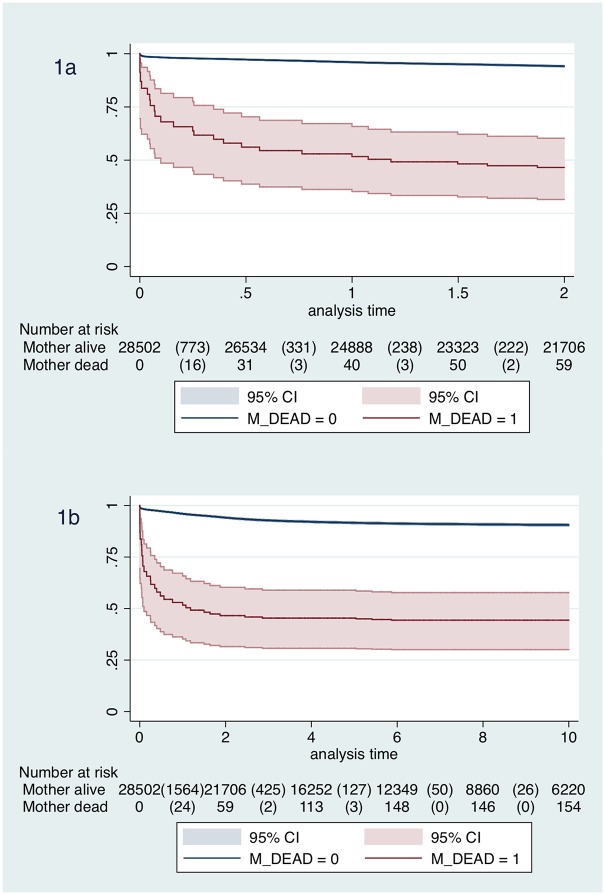
Kaplan-Meier survival estimates by maternal vital status for a) first 2-years, b) up to 10 years.

Child survival estimates by maternal vital status are shown in the Kaplan-Meier plots in Fig 1. Of the 2,221 deaths, 354 (15.9%) occurred in the first week of life; 112 (5.0%) between 1 week and <1 month of age; 323 (14.5%) between 1 and <6 months of age; 334 (15.0%) between 6 months to <1 year; 465 (20.9%) between 1 to <2 years and 633 (28.5%) > = 2 years to 10 years of age. The mortality rate decreased from 652.03 per 1,000 child-years (95% CI 587.53–723.62) in the first week of life to 6.15 per 1,000 child-years (95% CI 5.69–6.64) after 2 years of age. Overall mortality figures and rates for each of the 6 age categories and final adjusted hazard ratios for each child age category are presented in Table 2 (see also Table B in S1 File for the unadjusted analysis for each predictor variables in each age category). Maternal vital status was significantly associated to the risk of dying within the first 2 years of life (p-value <0.05), while this was no longer observed for children over 2 years of age (P = 0.872).

**Table 2:** Mortality rates and predictors for child death.

	Child deaths	Child-years	Rate per 1,000 child years	95% CI	HR*	95% CI
Overall	2,221	152,905.53	14.53	(13.93–15.14)		
Mother alive	2,192	151,785.35	14.44	(13.85–15.06)	1	
Mother dead	29	1,120.18	25.89	(17.99–37.25)	4.66	(3.15–6.89)
**Birth to <1 week**						
Mother alive	350	542.43	645.24	(581.06–716.51)	1	
Mother dead	4	0.49	8,222.23	(3,085.95–21,907.37)	3.05	(1.12–8.28)
Male	205	276.99	740.10	(645.41–848.67)	1	
Female	149	265.93	560.30	(477.19–657.89)	0.77	(0.62–0.95)
Year of birth						
1989–1995	78	73.59	1,059.98	(849.02–1,323.35)	1	
1996–2000	48	56.63	847.59	(638.74–1,124.72)	0.92	(0.61–1.39)
2001–2005	67	113.43	590.67	(464.89–750.47)	0.79	(0.54–1.15)
2006–2010	72	159.71	450.82	(357.84–567.96)	0.71	(0.49–1.02)
2011–2014	89	139.56	637.71	(518.08–784.97)	1.03	(0.73–1.45)
Rural	260	317.30	819.40	(725.62–925.31)	1	
Urban	94	225.62	416.64	(340.38–509.98)	0.51	(0.38–0.69)
Birth spacing to nearest sibling						
<18 months	158	48.65	3,247.87	(2,778.95–3,795.92)	1	
18–36 months	88	297.77	295.53	(239.81–364.20)	0.09	(0.07–0.12)
>36months	48	110.97	432.55	(325.97–573.98)	0.14	(0.10–0.20)
No sibling	60	85.53	701.51	(544.68–903.49)	0.26	(0.18–0.36)
**1 week to <1 month**						
Mother alive	107	1,791.59	59.72	(49.41–72.18)	1	
Mother dead	5	1.87	2,679.35	(1,115.22–6,437.23)	6.99	(2.98–16.36)
Year of birth						
1989–1995	28	242.88	115.28	(79.60–166.97)	1	
1996–2000	21	186.94	112.34	(73.25–172.30)	1.10	(0.62–1.93)
2001–2005	22	375.48	58.59	(38.58–88.99)	0.88	(0.52–1.50)
2006–2010	18	529.61	33.99	(21.41–53.94)	0.59	(0.33–1.05)
2011–2014	23	458.55	50.16	(33.33–75.48)	0.79	(0.45–1.39)
Rural	93	1,047.27	88.80	(72.47–108.82)	1	
Urban	19	746.19	25.46	(16.24–39.92)	0.40	(0.25–0.65)
Birth spacing to nearest sibling						
<18 months	59	156.42	377.19	(292.25–486.84)	1	
18–36 months	23	987.99	23.28	(15.47–35.03)	0.07	(0.05–0.12)
>36months	17	367.44	46.27	(28.76–74.42)	0.14	(0.09–0.24)
No sibling	13	281.61	46.16	(26.81–79.50)	0.19	(0.11–0.33)
**1 month to <6 months**						
Mother alive	316	11,314.77	27.93	(25.01–31.18)	1	
Mother dead	7	12.91	542.24	(258.51–1,137.42)	4.81	(2.30–10.06)
Year of birth						
1989–1995	88	1,552.37	56.69	(46.00–69.86)	1	
1996–2000	60	1,192.10	50.33	(39.08–64.82)	0.98	(0.70–1.35)
2001–2005	78	2,396.17	32.55	(26.07–40.64)	0.80	(0.58–1.12)
2006–2010	51	3,403.84	14.98	(11.39–19.71)	0.41	(0.29–0.59)
2011–2014	46	2,783.19	16.53	(12.38–22.07)	0.47	(0.32–0.69)
Rural	258	6,631.99	38.90	(34.43–43.95)	1	
Urban	65	4,695.69	13.84	(10.86–17.65)	0.51	(0.37–0.70)
Birth spacing to nearest sibling						
<18 months	124	962.09	128.89	(108.08–153.69)	1	
18–36 months	120	6,311.20	19.01	(15.90–22.74)	0.16	(0.12–0.21)
>36months	43	2,326.85	18.48	(13.71–24.92)	0.16	(0.11–0.23)
No sibling	36	1,727.53	20.84	(15.03–28.89)	0.22	(0.15–0.33)
**6 months to <1 year**						
Mother alive	331	12,877.01	25.70	(23.08–28.63)	1	
Mother dead	3	18.12	165.53	(53.39–513.24)	1.12	(0.23–5.35)
Year of birth						
1989–1995	92	1,811.28	50.79	(41.41–62.31)	1	
1996–2000	73	1,378.15	52.97	(42.11–66.63)	1.09	(0.79–1.48)
2001–2005	76	2,772.30	27.41	(21.89–34.33)	0.71	(0.51–1.00)
2006–2010	57	3,991.60	14.28	(11.01–18.51)	0.37	(0.26–0.53)
2011–2014	36	2,941.81	12.24	(8.83–16.97)	0.32	(0.21–0.49)
Rural	261	7,584.50	34.41	(30.48–38.85)	1	
Urban	73	5,310.64	13.75	(10.93–17.29)	0.65	(0.47–0.88)
Maternal age continuous					1.02	(1.01–1.03)
Birth spacing to nearest sibling						
<18 months	73	1,078.55	67.68	(53.81–85.14)	1	
18–36 months	174	7,323.71	23.76	(20.48–27.56)	0.38	(0.29–0.52)
>36months	44	2,659.08	16.55	(12.31–22.24)	0.26	(0.18–0.38)
No sibling	43	1,833.80	23.45	(17.39–31.62)	0.48	(0.32–0.72)
**1 year to <2 years**						
Mother alive	460	23,315.09	19.73	(18.01–21.62)	1	
Mother dead	5	50.74	98.54	(41.01–236.74)	3.63	(1.56–8.47)
Year of birth						
1989–1995	156	3,416.72	45.66	(39.03–53.42)	1	
1996–2000	104	2,594.65	40.08	(33.07–48.58)	0.40	(0.30–0.54)
2001–2005	81	5,277.51	15.35	(12.34–19.08)	0.30	(0.22–0.40)
2006–2010	84	7,663.99	10.96	(8.85–13.57)	0.25	(0.18–0.36)
2011–2014	40	4,412.95	9.06	(6.65–12.36)	0.00	(0.00–0.00)
Rural	375	13,952.62	26.88	(24.29–29.74)	1	
Urban	90	9,413.22	9.56	(7.78–11.76)	0.00	(0.00–0.00)
Birth spacing to nearest sibling						
<18 months	66	1,969.86	33.50	(26.32–42.65)	1	
18–36 months	289	13,694.76	21.10	(18.80–23.68)	0.71	(0.53–0.94)
>36months	60	4,862.05	12.34	(9.58–15.89)	0.42	(0.29–0.60)
No sibling	50	2,839.17	17.61	(13.35–23.24)	0.70	(0.48–1.03)
**> = 2 years**						
Mother alive	628	101,944.45	6.16	(5.70–6.66)	1	
Mother dead	5	1,036.06	4.83	(2.01–11.59)	0.93	(0.37–2.33)
Year of birth						
1989–1995	272	21,345.13	12.74	(11.32–14.35)	1	
1996–2000	147	15,962.10	9.21	(7.83–10.83)	0.72	(0.59–0.88)
2001–2005	108	33,003.65	3.27	(2.71–3.95)	0.30	(0.24–0.38)
2006–2010	96	29,513.76	3.25	(2.66–3.97)	0.22	(0.17–0.28)
2011–2014	10	3,155.87	3.17	(1.70–5.89)	0.11	(0.06–0.21)
Rural	541	69,020.38	7.84	(7.20–8.53)	1	
Urban	92	33,960.13	2.71	(2.21–3.32)	0.66	(0.51–0.85)
Birth spacing to nearest sibling						
<18 months	75	8,995.32	8.34	(6.65–10.46)	1	
18–36 months	383	63,048.76	6.07	(5.50–6.71)	0.78	(0.59–1.01)
>36months	128	23,271.76	5.50	(4.63–6.54)	0.73	(0.54–1.00)
No sibling	47	7,664.67	6.13	(4.61–8.16)	0.79	(0.53–1.16)

*Cox regression models run for each age category providing Hazard Ratios (HR) adjusted for each variable within the age category

Across the 6 different child age categories in the Cox regression models, children from the rural areas were at a higher risk of dying compared to those living in the more urban areas (Table 2). In the first week of life, girls were less likely to die (adjusted HR: 0.77, 95% CI: 0.62–0.95, P = 0.016), with a rate of 560.3 per 1,000 child-years (95%CI 477–658) compared to boys (740.0 per 1,000 child-years (CI: 645–849). The interaction between maternal vital status and sex was non-significant (P = 0.61) and there was no evidence for an association between gender and risk of death after 1 week of age. Year of birth was associated with lower risk of death for children older than 1 month of age and born after 2000. Birth spacing of less than 18 months to the nearest sibling was strongly associated with higher risk of death across the 6 child age groups.

**Fig 2:**
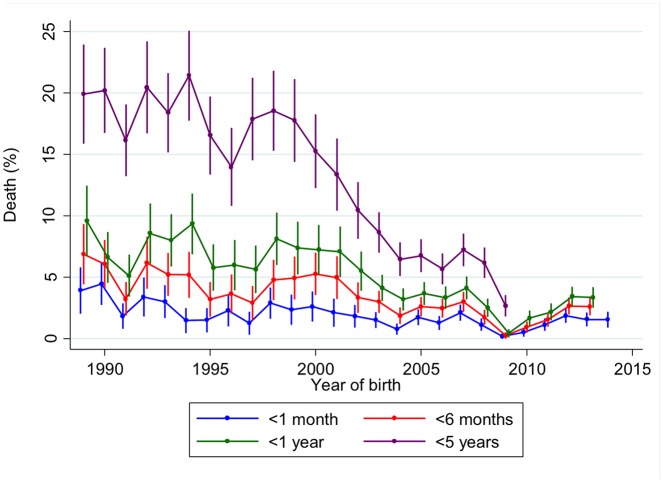
Percentage of deaths by age group and year of birth. Dip due to an interruption in surveillance between February 2008 and March 2009.

Fig 2 shows the percentage of deaths by age category and year of birth. Child mortality within the 1 to 5 year age group decreased from 19.9% (95%CI 15.9%-23.9%) in 1989 to 2.7% (95%CI 1.8%-3.6%) in 2009. Smaller but still significant declines were observed in 1–6 month and 7 month to 1 year age groups. However, there has been no change in the proportion of infants who died within the first month of life over that past 25 years. With only 29 of deaths among mothers occurring before their child’s death, it was not possible to further explore the relationship between year of birth and maternal vital status on child’s survival. However, nearly two-thirds of these deaths (n = 18) occurred before the year 2000.

## Discussion

Using one of the oldest demographic surveillance systems in sub-Saharan Africa, we observed that mother’s survival is strongly associated with child survival. Infants whose mothers died during delivery or shortly after were up to 7 times more likely to die within the first month of life than those whose mothers survived. This study is consistent with recent studies in East Africa [15, 16]. These infants lose the essential immediate mother-to-child contact care as well as vital nutrition from breast milk which has a drastic impact on their health and survival. Mortality rates within the first week of life, regardless of mother’s status, were very high, highlighting the increasing concern of neonatal survival in rural areas of sub-Saharan Africa. Many of these children are unable to receive the life-saving treatment they require due to the lack of access to appropriate care during delivery or adequate immediate postpartum management [17]. We observed the impact of a mother dying on child survival up to 2 years of age, after which the association is no longer observed and is consistent with a previous study in rural South Africa[7]. Other studies have continued to find an impact up to 5 years[8] and even 10 years of age[10]. The lack of impact after the child is 2 years may be due to the family structure in rural Gambia. This society is polygamous with strong extended families; grandmothers often live with the family and can be important replacement carers when the mother is no longer around[9]. Most severe illnesses due to diarrhoea and pneumonia occur within the first 2 years of life. It is possible that due to the relatively low incidence of these severe illnesses after 2 years of age, maternal care might have a lower impact on mortality outcome[18, 19]. All these studies, however, highlight the importance of caring for mothers’ beyond the early post-partum period. Maternal health remains important after pregnancy and delivery. The standard definition of maternal mortality is a death occurring during pregnancy or up to 42 days after termination of pregnancy. In the recently published global burden of disease study of levels and causes of maternal mortality during 1990–2013, 19% of the estimated maternal deaths in the sub-Saharan African Western region occurred during delivery and 49% between 24 hours and 6 weeks after delivery[20]. However, complications during this time period often do not end there and a significant proportion of women (16%) die due to pregnancy and delivery complications after this period[20]. WHO recommends contact with mothers at six weeks after delivery, but no routine checks are scheduled and this rarely occurs[21].

Newborn boys are at greater risk of death compared to females, a finding seen in previous studies[22]. Biological explanations include the impact of sex hormones on the immune system which can lead to greater susceptibility to and severity of infectious diseases in males but further studies are warranted[23]. Even in this largely rural area, we observed higher child mortality in the very rural remote areas compared to those in the urban areas. Farafenni town has one hospital, which consists of a 250-bed facility with paediatric, obstetric, medical, surgical, dental and ophthalmic units, as well as laboratories for haematology, biochemistry and parasitology. The town also has several private dispensaries and pharmacies. The more rural and remote areas are covered by 16 Primary Health Centre posts operated by village health workers under the supervision of community health nurses[14]. These health workers can only provide very basic health care, for example rapid diagnostic tests for malaria, and support referral processes. Although, place of delivery was not available in this data set, the recent demographic and health survey[24] estimated that 45% of deliveries in this region occurred at home with no skilled attendant. With 42% of women reporting that distance to a health facility was a key problem in accessing health care[24], we confirm that poor access to care and the level of care experienced in rural areas negatively impacts on child survival.

There have been numerous studies of infant and child mortality using DHS but few using longitudinal data in West Africa[8]. Such data sets benefit from data collected every four months over long periods of time and are thus able to show more accurate time trends and adjust for covariates. We show that overall child mortality has decreased over time and in particular since the initiation of the MDGs as reported in previous studies[12]. Such large reductions are largely due to several successful health programmes, such as improved vaccination coverage and the scaling up of intermittent preventive treatment for malaria among pregnant women and long lasting insecticidal nets[12, 14]. Our data are also consistent with the global trend of lacklustre improvements in neonatal mortality in the face of impressive reductions in overall child mortality, and re-emphasises the urgent need to find new ways to tackle this major public health concern. Within this surveillance system, strong efforts are made to capture all deaths. Within each village, there is a voluntary village reporter who also records all births and deaths within the village. Every 3–4 months, the DHSS field worker visits each house and routinely collects data on any births or deaths that may have occurred between the last and current survey. For any such event, these are then validated with the village reporter. However it is still possible that the number of deaths, and more specifically neonatal deaths[25], may have been underestimated, possibly resulting in an underestimation of the association.

We observed a weak association between mother’s age at the time of death and child survival. 16% of women who died before their child’s death were aged less than 20 years compared to only 1.5% of those whose child survived their death. Adolescent new mothers are considered a vulnerable population with high mortality and morbidity[26], but these data also indicate that their offspring are at high risk of adverse outcomes. Our study, found that women who left before the end of the study period were younger at time of their child’s birth compared to those who remained. It is thus possible that we have also missed younger maternal deaths and deaths of their offspring. Birth spacing remains an important risk factor for child death[8], which further highlights the importance of family planning and counselling soon after delivery. Only 9% of married women in The Gambia are using a contraceptive method and 25% of married women have an unmet need for family planning[24]. Family planning has been identified as a major intervention to improve health, but finding sustainable methods to improve coverage remains a challenge.

Our findings highlight the importance of the continuum of care for both the mother and child over time. Maternal mortality can be reduced with high quality skilled birth attendants and emergency obstetric care. However, care should not stop at delivery. The current integrated packages between maternal, newborn and child health are focused primarily around pre-conception, pregnancy, delivery, and post natal care for the newborn. Of the 142 reproductive, maternal, newborn and child health (RMNCH) interventions that have been identified and assessed, only 4 are directed to postnatal care of the mother. These are: family planning, anaemia measurements and treatment, post-natal sepsis (acute) and HIV screening and treatment. There are no recommended RMNCH interventions to assess long term and /or persistent complications post pregnancy and delivery[27] beyond 6 weeks post-partum.

Important progress has been made in reducing maternal mortality since the initiation of the MDGs. However, with the increase in survival of women who have complications during pregnancy and delivery, there may be a detrimental impact on the long term health of such women, as these women often have an increased risk of death[28]. The importance of interventions to fill the major gap in the continuum of care for maternal post-partum care has been highlighted in recent calls and several packages of care for the post natal period have been identified[21, 27, 29, 30]. As we move into the era of Sustainable Development Goals, integrating care for mother, newborn and child is essential. Thus, improving care of women during the inter-partum stage and beyond the 6 weeks post-delivery will also improve survival outcomes of her child.

## Supporting information

S1 File Table A in S1 File: Child Outcomes. Table B in S1 File: Mortality rates and Hazard ratio for risk of dying by general characteristics for each age category.
(DOCX)

## References

[1] Bustreo F, Say L, Koblinsky M, Pullum TW, Temmerman M, Pablos-Mendez A. Ending preventable maternal deaths: the time is now. The Lancet Global health. 2013;1(4):e176–7. Epub 2014/08/12. 10.1016/S2214-109X(13)70059-7 25104339

[2] Bhutta ZA, Das JK, Bahl R, Lawn JE, Salam RA, Paul VK, et al Can available interventions end preventable deaths in mothers, newborn babies, and stillbirths, and at what cost? Lancet. 2014;384(9940):347–70. Epub 2014/05/24. 10.1016/S0140-6736(14)60792-3 24853604

[3] Mason E, McDougall L, Lawn JE, Gupta A, Claeson M, Pillay Y, et al From evidence to action to deliver a healthy start for the next generation. Lancet. 2014;384(9941):455–67. Epub 2014/05/24. 10.1016/S0140-6736(14)60750-9 24853599

[4] Newell ML, Coovadia H, Cortina-Borja M, Rollins N, Gaillard P, Dabis F, et al Mortality of infected and uninfected infants born to HIV-infected mothers in Africa: a pooled analysis. Lancet. 2004;364(9441):1236–43. Epub 2004/10/07. 10.1016/S0140-6736(04)17140-7 15464184

[5] Taha TE, Miotti P, Liomba G, Dallabetta G, Chiphangwi J. HIV, maternal death and child survival in Africa. Aids. 1996;10(1):111–12. Epub 1996/01/01. 8924243 10.1097/00002030-199601000-00021

[6] Zaba B, Whitworth J, Marston M, Nakiyingi J, Ruberantwari A, Urassa M, et al HIV and mortality of mothers and children: evidence from cohort studies in Uganda, Tanzania, and Malawi. Epidemiology. 2005;16(3):275–80. Epub 2005/04/13. 15824540 10.1097/01.ede.0000155507.47884.43

[7] Clark SJ, Kahn K, Houle B, Arteche A, Collinson MA, Tollman SM, et al Young children's probability of dying before and after their mother's death: a rural South African population-based surveillance study. PLoS medicine. 2013;10(3):e1001409 Epub 2013/04/05. 10.1371/journal.pmed.1001409 23555200 PMC3608552

[8] Becher H, Muller O, Jahn A, Gbangou A, Kynast-Wolf G, Kouyate B. Risk factors of infant and child mortality in rural Burkina Faso. Bulletin of the World Health Organization. 2004;82(4):265–73. Epub 2004/07/21. 15259255 PMC2585959

[9] Sear R, Steele F, McGregor IA, Mace R. The effects of kin on child mortality in rural Gambia. Demography. 2002;39(1):43–63. Epub 2002/02/21. 11852839 10.1353/dem.2002.0010

[10] Ronsmans C, Chowdhury ME, Dasgupta SK, Ahmed A, Koblinsky M. Effect of parent's death on child survival in rural Bangladesh: a cohort study. Lancet. 2010;375(9730):2024–31. Epub 2010/06/24. 10.1016/S0140-6736(10)60704-0 20569842

[11] Scott S, Odutola A, Mackenzie G, Fulford T, Afolabi MO, Lowe Jallow Y, et al Coverage and timing of children's vaccination: an evaluation of the expanded programme on immunisation in The Gambia. PloS one. 2014;9(9):e107280 Epub 2014/09/19. 10.1371/journal.pone.0107280 25232830 PMC4169419

[12] Jasseh M, Webb EL, Jaffar S, Howie S, Townend J, Smith PG, et al Reaching millennium development goal 4—the Gambia. Tropical medicine & international health: TM & IH. 2011;16(10):1314–25. Epub 2011/06/29. 21707875 10.1111/j.1365-3156.2011.02809.x

[13] Streatfield PK, Alam N, Compaore Y, Rossier C, Soura AB, Bonfoh B, et al Pregnancy-related mortality in Africa and Asia: evidence from INDEPTH Health and Demographic Surveillance System sites. Global health action. 2014;7:25368 Epub 2014/11/08. 10.3402/gha.v7.25368 25377328 PMC4220143

[14] Jasseh M, Gomez P, Greenwood BM, Howie SR, Scott S, Snell PC, et al Health & Demographic Surveillance System Profile: Farafenni Health and Demographic Surveillance System in The Gambia. International journal of epidemiology. 2015. Epub 2015/05/08. 10.1093/ije/dyv049 25948661

[15] Moucheraud C, Worku A, Molla M, Finlay JE, Leaning J, Yamin A. Consequences of maternal mortality on infant and child survival: a 25-year longitudinal analysis in Butajira Ethiopia (1987–2011). Reproductive health. 2015;12 Suppl 1:S4. 10.1186/1742-4755-12-S1-S4 PMC4423767 26001059

[16] Finlay JE, Moucheraud C, Goshev S, Levira F, Mrema S, Canning D, et al The Effects of Maternal Mortality on Infant and Child Survival in Rural Tanzania: A Cohort Study. Maternal and child health journal. 2015;19(11):2393–402. 10.1007/s10995-015-1758-2 26100131

[17] de Bernis L, Sherratt DR, AbouZahr C, van Lerberghe W. Skilled attendants for pregnancy, childbirth and postnatal care. Br Med Bull. 2003;67:39–57. 14711753 10.1093/bmb/ldg017

[18] Cutts FT, Zaman SM, Enwere G, Jaffar S, Levine OS, Okoko JB, et al Efficacy of nine-valent pneumococcal conjugate vaccine against pneumonia and invasive pneumococcal disease in The Gambia: randomised, double-blind, placebo-controlled trial. Lancet. 2005;365(9465):1139–46. 10.1016/S0140-6736(05)71876-6 15794968

[19] Kotloff KL, Nataro JP, Blackwelder WC, Nasrin D, Farag TH, Panchalingam S, et al Burden and aetiology of diarrhoeal disease in infants and young children in developing countries (the Global Enteric Multicenter Study, GEMS): a prospective, case-control study. Lancet. 2013;382(9888):209–22. 10.1016/S0140-6736(13)60844-2 23680352

[20] Kassebaum NJ, Bertozzi-Villa A, Coggeshall MS, Shackelford KA, Steiner C, Heuton KR, et al Global, regional, and national levels and causes of maternal mortality during 1990–2013: a systematic analysis for the Global Burden of Disease Study 2013. Lancet. 2014;384(9947):980–1004. Epub 2014/05/07. 10.1016/S0140-6736(14)60696-6 24797575 PMC4255481

[21] World Health Organisation. WHO recommendations on postnatal care of the mother and newborn. Geneva, Switzerland: 2013. 24624481

[22] Lawn JE, Blencowe H, Oza S, You D, Lee AC, Waiswa P, et al Every Newborn: progress, priorities, and potential beyond survival. Lancet. 2014;384(9938):189–205. 10.1016/S0140-6736(14)60496-7 24853593

[23] Muenchhoff M, Goulder P. Sex Differences in Pediatric Infectious Diseases. The Journal of infectious diseases. 2014;209(S3):S120–6. 24966192 10.1093/infdis/jiu232 PMC4072001

[24] The Gambia Bureau of Statistics (GBOS) and ICF International. The Gambia Demographic and Health Survey 2013. Banjul, The Gambia and Rockvilla, Maryland, USA: GBOS and ICF International: 2014.

[25] Jasseh M, Howie SR, Gomez P, Scott S, Roca A, Cham M, et al Disease-specific mortality burdens in a rural Gambian population using verbal autopsy, 1998–2007. Global health action. 2014;7:25598 Epub 2014/11/08. 10.3402/gha.v7.25598 25377344 PMC4220164

[26] WHO Guidelines on Preventing Early Pregnancy and Poor Reproductive Health Outcomes Among Adolescents in Developing Countries WHO Guidelines Approved by the Guidelines Review Committee. Geneva 2011. 26180870

[27] The Partnership for Maternal Newborn & Child Health. A Global Review of the Key Interventions Related to Reproductive, Maternal, Newborn and Child Health (RMNCH)). Geneva, Switzerland: 2011.

[28] Storeng KT, Drabo S, Ganaba R, Sundby J, Calvert C, Filippi V. Mortality after near-miss obstetric complications in Burkina Faso: medical, social and health-care factors. Bulletin of the World Health Organization. 2012;90(6):418–25B. Epub 2012/06/13. 10.2471/BLT.11.094011 22690031 PMC3370364

[29] Bhutta ZA, Darmstadt GL, Hasan BS, Haws RA. Community-based interventions for improving perinatal and neonatal health outcomes in developing countries: a review of the evidence. Pediatrics. 2005;115(2 Suppl):519–617. Epub 2005/05/04. 10.1542/peds.2004-1441 15866863

[30] Lassi ZS, Kumar R, Mansoor T, Salam RA, Das JK, Bhutta ZA. Essential interventions: implementation strategies and proposed packages of care. Reproductive health. 2014;11 Suppl 1:S5. Epub 2014/09/02. 10.1186/1742-4755-11-S1-S5 PMC4145859 25178110

