# Supplementary material for: Effect of maternal death on child survival in rural West Africa: 25 years of prospective surveillance data in The Gambia
Source: PLoS One. 2017 Feb 22;12(2):e0172286. doi: 10.1371/journal.pone.0172286 (PMC5321282; doi:10.1371/journal.pone.0172286)
Supplement: S1 File — Table A in S1 File: Child Outcomes. Table B in S1 File: Mortality rates and Hazard ratio for risk of dying by general characteristics for each age category. (DOCX) [file pone.0172286.s001.docx]

**S1 File**

**Table A: Child outcomes**

| Child's status | Number | % |
| --- | --- | --- |
| Alive at end of follow up | 21,248 | 74.5% |
| Deaths | 2,221 | 7.8% |
| Exit during study | 5,033 | 17.7% |
| Total | 28,502 |  |

**Table B: Mortality rates and Hazard ratio for risk of dying by general characteristics for each age category**

|  | **Child deaths** | **Child-years** | **Rate per 1,000 child years** | **95% CI** | **Hazard Ratio** | **95% CI** |
| --- | --- | --- | --- | --- | --- | --- |
| **Overall** | 2,221 | 152,905.5 | 14.53 | (13.93-15.14) |  |  |
| birth to <1 week | 354 | 542.9 | 652.03 | (587.53-723.62) |  |  |
| 1 week to <1 month | 112 | 1,793.5 | 62.45 | (51.89-75.16) |  |  |
| 1 month to <6 months | 323 | 11,327.7 | 28.51 | (25.57-31.80) |  |  |
| 6 months to <1 year | 334 | 12,895.1 | 25.90 | (23.27-28.83) |  |  |
| 1 year to <2 years | 465 | 23,365.8 | 19.90 | (18.17-21.79) |  |  |
| >=2 years | 633 | 102,980.5 | 6.15 | (5.69-6.64) |  |  |
|  |  |  |  |  |  |  |
| **Mother's vital status** |  |  |  |  |  |  |
| Mother alive | 2,192 | 151,785.4 | 14.44 | (13.85-15.06) |  |  |
| birth to <1 week | 350 | 542.4 | 645.24 | (581.06-716.51) | 1 |  |
| 1 week to <1 month | 107 | 1,791.6 | 59.72 | (49.41-72.18) | 1 |  |
| 1 month to <6 months | 316 | 11,314.8 | 27.93 | (25.01-31.18) | 1 |  |
| 6 months to <1 year | 331 | 12,877.0 | 25.70 | (23.08-28.63) | 1 |  |
| 1 year to <2 years | 460 | 23,315.1 | 19.73 | (18.01-21.62) | 1 |  |
| 2-10 years | 628 | 101,944.5 | 6.16 | (5.70-6.66) | 1 |  |
|  |  |  |  |  |  |  |
| Mother dead | 29 | 1,120.2 | 25.89 | (17.99-37.25) |  |  |
| birth to <1 week | 4 | 0.5 | 8222.23 | (3,085.95-21,907.37) | 14.47 | (5.64-37.08) |
| 1 week to <1 month | 5 | 1.9 | 2679.35 | (1,115.22-6,437.23) | 39.61 | (17.21-91.17) |
| 1 month to <6 months | 7 | 12.9 | 542.24 | (258.51-1,137.42) | 19.87 | (9.43-41.87) |
| 6 months to <1 year | 3 | 18.1 | 165.53 | (53.39-513.24) | 4.33 | (1.06-17.64) |
| 1 year to <2 years | 5 | 50.7 | 98.54 | (41.01-236.74) | 6.01 | (2.64-13.68) |
| 2-10 years | 5 | 1,036.1 | 4.83 | (2.01-11.59) | 1.21 | (0.50-2.94) |
|  |  |  |  |  |  |  |
| **Gender** |  |  |  |  |  |  |
| Male | 1,177 | 78,100.4 | 15.07 | (14.23-15.96) |  |  |
| birth to <1 week | 205 | 277.0 | 740.10 | (645.41-848.67) | 1 |  |
| 1 week to <1 month | 57 | 914.3 | 62.34 | (48.09-80.82) | 1 |  |
| 1 month to <6 months | 165 | 5,771.3 | 28.59 | (24.54-33.30) | 1 |  |
| 6 months to <1 year | 186 | 6,565.6 | 28.33 | (24.54-32.71) | 1 |  |
| 1 year to <2 years | 236 | 11,879.3 | 19.87 | (17.49-22.57) | 1 |  |
| 2-10 years | 328 | 52,692.9 | 6.22 | (5.59-6.94) | 1 |  |
|  |  |  |  |  |  |  |
| Female | 1,044 | 74,805.1 | 13.96 | (13.13-14.83) |  |  |
| birth to <1 week | 149 | 265.9 | 560.30 | (477.19-657.89) | 0.75 | (0.60-0.93) |
| 1 week to <1 month | 55 | 879.1 | 62.56 | (48.03-81.49) | 1.01 | (0.71-1.43) |
| 1 month to <6 months | 158 | 5,556.4 | 28.44 | (24.33-33.23) | 0.99 | (0.79-1.22) |
| 6 months to <1 year | 148 | 6,329.6 | 23.38 | (19.90-27.47) | 0.84 | (0.67-1.05) |
| 1 year to <2 years | 229 | 11,486.5 | 19.94 | (17.51-22.69) | 1.00 | (0.83-1.19) |
| 2-10 years | 305 | 50,287.6 | 6.07 | (5.42-6.79) | 0.98 | (0.84-1.14) |
|  |  |  |  |  |  |  |
| **Ethnic group** |  |  |  |  |  |  |
| Wollof | 987 | 64,383.3 | 15.33 | (14.40-16.32) |  |  |
| birth to <1 week | 137 | 235.7 | 581.36 | (491.72-687.33) | 1 |  |
| 1 week to <1 month | 44 | 778.8 | 56.50 | (42.04-75.92) | 1 |  |
| 1 month to <6 months | 146 | 4,915.7 | 29.70 | (25.25-34.93) | 1 |  |
| 6 months to <1 year | 159 | 5,600.0 | 28.39 | (24.31-33.17) | 1 |  |
| 1 year to <2 years | 210 | 10,116.1 | 20.76 | (18.13-23.77) | 1 |  |
| 2-10 years | 291 | 42,737.1 | 6.81 | (6.07-7.64) | 1 |  |
|  |  |  |  |  |  |  |
| Mandinka | 720 | 50,253.7 | 14.33 | (13.32-15.41) |  |  |
| birth to <1 week | 138 | 171.4 | 804.96 | (681.26-951.12) | 1.46 | (1.12-1.91) |
| 1 week to <1 month | 42 | 565.9 | 74.22 | (54.85-100.42) | 1.20 | (0.81-1.78) |
| 1 month to <6 months | 100 | 3,576.6 | 27.96 | (22.98-34.01) | 0.95 | (0.73-1.23) |
| 6 months to <1 year | 107 | 4,079.6 | 26.23 | (21.70-31.70) | 0.94 | (0.73-1.21) |
| 1 year to <2 years | 143 | 7,448.8 | 19.20 | (16.30-22.62) | 0.91 | (0.73-1.13) |
| 2-10 years | 190 | 34,411.4 | 5.52 | (4.79-6.37) | 0.84 | (0.70-1.02) |
|  |  |  |  |  |  |  |
| Fula | 468 | 33,783.5 | 13.85 | (12.65-15.17) |  |  |
| birth to <1 week | 71 | 117.3 | 605.43 | (479.79-763.99) | 1.12 | (0.82-1.54) |
| 1 week to <1 month | 24 | 387.2 | 61.98 | (41.54-92.47) | 0.96 | (0.59-1.56) |
| 1 month to <6 months | 71 | 2,450.3 | 28.98 | (22.96-36.56) | 0.99 | (0.74-1.32) |
| 6 months to <1 year | 58 | 2,785.6 | 20.82 | (16.10-26.93) | 0.75 | (0.55-1.03) |
| 1 year to <2 years | 105 | 5,049.0 | 20.80 | (17.18-25.18) | 0.99 | (0.78-1.26) |
| 2-10 years | 139 | 22,994.0 | 6.05 | (5.12-7.14) | 0.92 | (0.74-1.14) |
|  |  |  |  |  |  |  |
| Other | 46 | 4,485.0 | 10.26 | (7.68-13.69) |  |  |
| birth to <1 week | 8 | 18.6 | 431.14 | (215.61-862.11) | 0.71 | (0.33-1.53) |
| 1 week to <1 month | 2 | 61.5 | 32.52 | (8.13-130.02) | 0.74 | (0.23-2.38) |
| 1 month to <6 months | 6 | 385.0 | 15.58 | (7.00-34.69) | 0.54 | (0.24-1.22) |
| 6 months to <1 year | 10 | 429.9 | 23.26 | (12.52-43.23) | 0.85 | (0.42-1.69) |
| 1 year to <2 years | 7 | 751.9 | 9.31 | (4.44-19.53) | 0.44 | (0.21-0.94) |
| 2-10 years | 13 | 2,838.1 | 4.58 | (2.66-7.89) | 0.64 | (0.37-1.12) |
|  |  |  |  |  |  |  |
| **Year of birth** |  |  |  |  |  |  |
| 1989-1995 | 714 | 28,442.0 | 25.10 | (23.33-27.01) |  |  |
| birth to <1 week | 78 | 73.6 | 1059.98 | (849.02-1,323.35) | 1 |  |
| 1 week to <1 month | 28 | 242.9 | 115.28 | (79.60-166.97) | 1 |  |
| 1 month to <6 months | 88 | 1,552.4 | 56.69 | (46.00-69.86) | 1 |  |
| 6 months to <1 year | 92 | 1,811.3 | 50.79 | (41.41-62.31) | 1 |  |
| 1 year to <2 years | 156 | 3,416.7 | 45.66 | (39.03-53.42) | 1 |  |
| 2-10 years | 272 | 21,345.1 | 12.74 | (11.32-14.35) | 1 |  |
|  |  |  |  |  |  |  |
| 1996-2000 | 453 | 21,370.6 | 21.20 | (19.33-23.24) |  |  |
| birth to <1 week | 48 | 56.6 | 847.59 | (638.74-1,124.72) | 0.79 | (0.52-1.20) |
| 1 week to <1 month | 21 | 186.9 | 112.34 | (73.25-172.30) | 0.93 | (0.53-1.64) |
| 1 month to <6 months | 60 | 1,192.1 | 50.33 | (39.08-64.82) | 0.88 | (0.64-1.22) |
| 6 months to <1 year | 73 | 1,378.1 | 52.97 | (42.11-66.63) | 1.01 | (0.75-1.38) |
| 1 year to <2 years | 104 | 2,594.6 | 40.08 | (33.07-48.58) | 0.91 | (0.72-1.16) |
| 2-10 years | 147 | 15,962.1 | 9.21 | (7.83-10.83) | 0.72 | (0.59-0.88) |
|  |  |  |  |  |  |  |
| 2001-2005 | 432 | 43,938.5 | 9.83 | (8.95-10.80) |  |  |
| birth to <1 week | 67 | 113.4 | 590.67 | (464.89-750.47) | 0.54 | (0.37-0.77) |
| 1 week to <1 month | 22 | 375.5 | 58.59 | (38.58-88.99) | 0.56 | (0.34-0.93) |
| 1 month to <6 months | 78 | 2,396.2 | 32.55 | (26.07-40.64) | 0.56 | (0.41-0.77) |
| 6 months to <1 year | 76 | 2,772.3 | 27.41 | (21.89-34.33) | 0.55 | (0.41-0.76) |
| 1 year to <2 years | 81 | 5,277.5 | 15.35 | (12.34-19.08) | 0.34 | (0.26-0.44) |
| 2-10 years | 108 | 33,003.6 | 3.27 | (2.71-3.95) | 0.25 | (0.20-0.32) |
|  |  |  |  |  |  |  |
| 2006-2010 | 378 | 45,262.5 | 8.35 | (7.55-9.24) |  |  |
| birth to <1 week | 72 | 159.7 | 450.82 | (357.84-567.96) | 0.42 | (0.29-0.59) |
| 1 week to <1 month | 18 | 529.6 | 33.99 | (21.41-53.94) | 0.32 | (0.19-0.55) |
| 1 month to <6 months | 51 | 3,403.8 | 14.98 | (11.39-19.71) | 0.26 | (0.19-0.37) |
| 6 months to <1 year | 57 | 3,991.6 | 14.28 | (11.01-18.51) | 0.28 | (0.20-0.39) |
| 1 year to <2 years | 84 | 7,664.0 | 10.96 | (8.85-13.57) | 0.24 | (0.19-0.32) |
| 2-10 years | 96 | 29,513.8 | 3.25 | (2.66-3.97) | 0.18 | (0.14-0.22) |
|  |  |  |  |  |  |  |
| 2011-2014 | 244 | 13,891.9 | 17.56 | (15.49-19.91) |  |  |
| birth to <1 week | 89 | 139.6 | 637.71 | (518.08-784.97) | 0.61 | (0.44-0.85) |
| 1 week to <1 month | 23 | 458.6 | 50.16 | (33.33-75.48) | 0.42 | (0.25-0.71) |
| 1 month to <6 months | 46 | 2,783.2 | 16.53 | (12.38-22.07) | 0.29 | (0.20-0.41) |
| 6 months to <1 year | 36 | 2,941.8 | 12.24 | (8.83-16.97) | 0.24 | (0.16-0.35) |
| 1 year to <2 years | 40 | 4,413.0 | 9.06 | (6.65-12.36) | 0.20 | (0.14-0.29) |
| 2-10 years | 10 | 3,155.9 | 3.17 | (1.70-5.89) | 0.09 | (0.05-0.17) |
|  |  |  |  |  |  |  |
| **Maternal age at birth** |  |  |  |  |  |  |
| <20 | 353 | 21,461.5 | 16.45 | (14.82-18.26) |  |  |
| birth to <1 week | 73 | 78.5 | 929.46 | (738.93-1,169.11) | 1 |  |
| 1 week to <1 month | 26 | 258.7 | 100.49 | (68.42-147.58) | 1 |  |
| 1 month to <6 months | 58 | 1,629.3 | 35.60 | (27.52-46.05) | 1 |  |
| 6 months to <1 year | 50 | 1,837.0 | 27.22 | (20.63-35.91) | 1 |  |
| 1 year to <2 years | 62 | 3,277.5 | 18.92 | (14.75-24.26) | 1 |  |
| 2-10 years | 84 | 14,380.4 | 5.84 | (4.72-7.23) | 1 |  |
|  |  |  |  |  |  |  |
| 21-30 | 947 | 75,460.7 | 12.55 | (11.78-13.37) |  |  |
| birth to <1 week | 136 | 272.9 | 498.39 | (421.29-589.61) | 0.55 | (0.40-0.76) |
| 1 week to <1 month | 35 | 902.8 | 38.77 | (27.84-54.00) | 0.38 | (0.24-0.61) |
| 1 month to <6 months | 141 | 5,697.9 | 24.75 | (20.98-29.19) | 0.69 | (0.50-0.94) |
| 6 months to <1 year | 138 | 6,485.1 | 21.28 | (18.01-25.14) | 0.77 | (0.55-1.07) |
| 1 year to <2 years | 213 | 11,742.4 | 18.14 | (15.86-20.75) | 0.98 | (0.74-1.30) |
| 2-10 years | 284 | 50,359.7 | 5.64 | (5.02-6.33) | 0.94 | (0.73-1.20) |
|  |  |  |  |  |  |  |
| 31-45 | 866 | 53,684.6 | 16.13 | (15.09-17.24) |  |  |
| birth to <1 week | 137 | 184.3 | 743.50 | (628.87-879.03) | 0.80 | (0.58-1.11) |
| 1 week to <1 month | 44 | 608.2 | 72.35 | (53.84-97.22) | 0.70 | (0.44-1.10) |
| 1 month to <6 months | 113 | 3,851.8 | 29.34 | (24.40-35.28) | 0.80 | (0.58-1.10) |
| 6 months to <1 year | 136 | 4,401.3 | 30.90 | (26.12-36.55) | 1.16 | (0.84-1.62) |
| 1 year to <2 years | 181 | 8,031.9 | 22.54 | (19.48-26.07) | 1.19 | (0.89-1.60) |
| 2-10 years | 255 | 36,607.0 | 6.97 | (6.16-7.88) | 1.18 | (0.92-1.52) |
|  |  |  |  |  |  |  |
| 45+ | 55 | 2,298.7 | 23.93 | (18.37-31.16) |  |  |
| birth to <1 week | 8 | 7.2 | 1105.01 | (552.61-2,209.59) | 1.27 | (0.61-2.67) |
| 1 week to <1 month | 7 | 23.8 | 294.56 | (140.43-617.86) | 2.61 | (1.15-5.92) |
| 1 month to <6 months | 11 | 148.6 | 74.01 | (40.99-133.65) | 1.89 | (0.98-3.64) |
| 6 months to <1 year | 10 | 171.7 | 58.23 | (31.33-108.23) | 2.11 | (1.06-4.19) |
| 1 year to <2 years | 9 | 314.0 | 28.67 | (14.92-55.09) | 1.39 | (0.64-2.99) |
| 2-10 years | 10 | 1,633.4 | 6.12 | (3.29-11.38) | 1.09 | (0.57-2.09) |
| **Continuous** |  |  |  |  |  |  |
| birth to <1 week |  |  |  |  | 1.01 | (0.99-1.03) |
| 1 week to <1 month |  |  |  |  | 1.02 | (0.99-1.05) |
| 1 month to <6 months |  |  |  |  | 1.01 | (0.99-1.02) |
| 6 months to <1 year |  |  |  |  | 1.02 | (1.01-1.04) |
| 1 year to <2 years |  |  |  |  | 1.00 | (0.99-1.02) |
| 2-10 years |  |  |  |  | 1.01 | (1.00-1.02) |
|  |  |  |  |  |  |  |
| **Region** |  |  |  |  |  |  |
| Rural | 1,788 | 98,554.1 | 18.14 | (17.32-19.00) |  |  |
| birth to <1 week | 260 | 317.3 | 819.40 | (725.62-925.31) | 1 |  |
| 1 week to <1 month | 93 | 1,047.3 | 88.80 | (72.47-108.82) | 1 |  |
| 1 month to <6 months | 258 | 6,632.0 | 38.90 | (34.43-43.95) | 1 |  |
| 6 months to <1 year | 261 | 7,584.5 | 34.41 | (30.48-38.85) | 1 |  |
| 1 year to <2 years | 375 | 13,952.6 | 26.88 | (24.29-29.74) | 1 |  |
| 2-10 years | 541 | 69,020.4 | 7.84 | (7.20-8.53) | 1 |  |
|  |  |  |  |  |  |  |
| Urban | 433 | 54,351.5 | 7.97 | (7.25-8.75) |  |  |
| birth to <1 week | 94 | 225.6 | 416.64 | (340.38-509.98) | 0.50 | (0.38-0.65) |
| 1 week to <1 month | 19 | 46.2 | 25.46 | (16.24-39.92) | 0.35 | (0.22-0.54) |
| 1 month to <6 months | 65 | 4,695.7 | 13.84 | (10.86-17.65) | 0.36 | (0.27-0.47) |
| 6 months to <1 year | 73 | 5,310.6 | 13.75 | (10.93-17.29) | 0.40 | (0.31-0.53) |
| 1 year to <2 years | 90 | 9,413.2 | 9.56 | (7.78-11.76) | 0.36 | (0.28-0.45) |
| 2-10 years | 92 | 33,960.1 | 2.71 | (2.21-3.32) | 0.30 | (0.24-0.37) |
|  |  |  |  |  |  |  |
| **Birth Order** |  |  |  |  |  |  |
| 1 | 872 | 61,761.8 | 14.12 | (13.21-15.09) |  |  |
| 2 | 532 | 36,687.7 | 14.50 | (13.32-15.79) |  |  |
| 3 | 332 | 22,688.7 | 14.63 | (13.14-16.29) |  |  |
| 4 | 232 | 13,564.9 | 17.10 | (15.04-19.45) |  |  |
| 5 | 127 | 8,227.8 | 15.44 | (12.97-18.37) |  |  |
| 6+ | 126 | 9,974.6 | 12.63 | (10.61-15.04) |  |  |
| **Continuous** |  |  |  |  |  |  |
| Overall |  |  |  |  | 0.99 | (0.96-1.01) |
| birth to <1 week |  |  |  |  | 0.98 | (0.92-1.05) |
| 1 week to <1 month |  |  |  |  | 0.99 | (0.91-1.08) |
| 1 month to <6 months |  |  |  |  | 1.00 | (0.94-1.06) |
| 6 months to <1 year |  |  |  |  | 1.07 | (1.01-1.13) |
| 1 year to <2 years |  |  |  |  | 0.99 | (0.93-1.04) |
| 2-10 years |  |  |  |  | 0.92 | (0.87-0.97) |
|  |  |  |  |  |  |  |
| **Birth spacing with closest younger sibling** | | |  |  |  |  |
| <18 months | 212 | 7,054.7 | 30.05 | (26.27-34.38) |  |  |
| birth to <1 week | 54 | 22.3 | 2416.29 | (1,850.61-3,154.88) | 1 |  |
| 1 week to <1 month | 20 | 72.9 | 274.28 | (176.95-425.14) | 1 |  |
| 1 month to <6 months | 32 | 457.3 | 69.98 | (49.49-98.95) | 1 |  |
| 6 months to <1 year | 32 | 519.8 | 61.56 | (43.54-87.06) | 1 |  |
| 1 year to <2 years | 31 | 955.2 | 32.45 | (22.82-46.15) | 1 |  |
| 2-10 years | 43 | 5,027.2 | 8.55 | (6.34-11.53) | 1 |  |
|  |  |  |  |  |  |  |
| 18-36 months | 743 | 56,836.8 | 13.07 | (12.17-14.05) |  |  |
| birth to <1 week | 88 | 203.6 | 432.12 | (350.64-532.53) | 0.18 | (0.13-0.26) |
| 1 week to <1 month | 28 | 673.8 | 41.55 | (28.69-60.18) | 0.22 | (0.12-0.40) |
| 1 month to <6 months | 103 | 4,271.4 | 24.11 | (19.88-29.25) | 0.43 | (0.27-0.67) |
| 6 months to <1 year | 133 | 4,887.3 | 27.21 | (22.96-32.25) | 0.46 | (0.30-0.69) |
| 1 year to <2 years | 169 | 8,900.2 | 18.99 | (16.33-22.08) | 0.67 | (0.45-1.01) |
| 2-10 years | 222 | 37,900.4 | 5.86 | (5.14-6.68) | 0.60 | (0.43-0.85) |
|  |  |  |  |  |  |  |
| >36 months | 394 | 27,252.2 | 14.46 | (13.10-15.96) |  |  |
| birth to <1 week | 54 | 103.0 | 524.50 | (401.71-684.82) | 0.21 | (0.14-0.32) |
| 1 week to <1 month | 31 | 339.8 | 91.22 | (64.15-129.72) | 0.49 | (0.27-0.89) |
| 1 month to <6 months | 59 | 2,140.2 | 27.57 | (21.36-35.58) | 0.48 | (0.30-0.78) |
| 6 months to <1 year | 55 | 2,428.2 | 22.65 | (17.39-29.50) | 0.40 | (0.25-0.63) |
| 1 year to <2 years | 89 | 4,378.9 | 20.32 | (16.51-25.02) | 0.71 | (0.46-1.09) |
| 2-10 years | 106 | 17,862.2 | 5.93 | (4.91-7.18) | 0.60 | (0.42-0.88) |
|  |  |  |  |  |  |  |
| No older sibling | 872 | 61,761.8 | 14.12 | (13.21-15.09) |  |  |
| birth to <1 week | 158 | 214.0 | 738.43 | (631.81-863.03) | 0.31 | (0.23-0.42) |
| 1 week to <1 month | 33 | 706.9 | 46.68 | (33.19-65.67) | 0.28 | (0.16-0.51) |
| 1 month to <6 months | 129 | 4,458.8 | 28.93 | (24.35-34.38) | 0.51 | (0.33-0.80) |
| 6 months to <1 year | 114 | 5,059.9 | 22.53 | (18.75-27.07) | 0.38 | (0.25-0.58) |
| 1 year to <2 years | 176 | 9,131.6 | 19.27 | (16.63-22.34) | 0.67 | (0.45-1.01) |
| 2-10 years | 262 | 42,190.7 | 6.21 | (5.50-7.01) | 0.67 | (0.48-0.94) |
|  |  |  |  |  |  |  |
| **Birth spacing with closest older sibling** | | |  |  |  |  |
| <18 months | 376 | 6,465.0 | 58.16 | (52.57-64.35) |  |  |
| birth to <1 week | 119 | 27.6 | 4306.01 | (3,597.87-5,153.53) | 1 |  |
| 1 week to <1 month | 42 | 87.5 | 479.87 | (354.63-649.33) | 1 |  |
| 1 month to <6 months | 98 | 528.6 | 185.41 | (152.11-226.01) | 1 |  |
| 6 months to <1 year | 43 | 585.1 | 73.49 | (54.50-99.09) | 1 |  |
| 1 year to <2 years | 39 | 1,065.5 | 36.60 | (26.74-50.10) | 1 |  |
| 2-10 years | 35 | 4,170.7 | 8.39 | (6.03-11.69) | 1 |  |
|  |  |  |  |  |  |  |
| 18-36 months | 853 | 71,134.3 | 11.99 | (11.21-12.82) |  |  |
| birth to <1 week | 63 | 202.2 | 311.59 | (243.42-398.87) | 0.08 | (0.06-0.10) |
| 1 week to <1 month | 17 | 672.0 | 25.30 | (15.73-40.69) | 0.06 | (0.03-0.10) |
| 1 month to <6 months | 91 | 4,342.1 | 20.96 | (17.07-25.74) | 0.12 | (0.09-0.15) |
| 6 months to <1 year | 149 | 5,147.3 | 28.95 | (24.65-33.99) | 0.40 | (0.29-0.57) |
| 1 year to <2 years | 231 | 10,054.0 | 22.98 | (20.20-26.14) | 0.63 | (0.45-0.88) |
| 2-10 years | 302 | 50,716.8 | 5.95 | (5.32-6.67) | 0.78 | (0.55-1.10) |
|  |  |  |  |  |  |  |
| >36 months | 374 | 40,352.7 | 9.27 | (8.37-10.26) |  |  |
| birth to <1 week | 33 | 102.4 | 322.19 | (229.05-453.20) | 0.08 | (0.06-0.12) |
| 1 week to <1 month | 10 | 340.4 | 29.38 | (15.81-54.61) | 0.06 | (0.03-0.11) |
| 1 month to <6 months | 37 | 2,201.9 | 16.80 | (12.18-23.19) | 0.09 | (0.06-0.14) |
| 6 months to <1 year | 43 | 2,616.0 | 16.44 | (12.19-22.16) | 0.22 | (0.14-0.34) |
| 1 year to <2 years | 69 | 5,146.7 | 13.41 | (10.59-16.97) | 0.37 | (0.25-0.55) |
| 2-10 years | 182 | 29,945.3 | 6.08 | (5.26-7.03) | 0.84 | (0.59-1.19) |
|  |  |  |  |  |  |  |
| No older sibling | 618 | 34,953.5 | 17.68 | (16.34-19.13) |  |  |
| birth to <1 week | 139 | 210.7 | 659.79 | (558.74-779.12) | 0.16 | (0.12-0.20) |
| 1 week to <1 month | 43 | 693.6 | 62.00 | (45.98-83.59) | 0.13 | (0.09-0.19) |
| 1 month to <6 months | 97 | 4,255.2 | 22.80 | (18.68-27.81) | 0.12 | (0.09-0.16) |
| 6 months to <1 year | 99 | 4,546.7 | 21.77 | (17.88-26.51) | 0.30 | (0.21-0.43) |
| 1 year to <2 years | 126 | 7,099.6 | 17.75 | (14.90-21.13) | 0.47 | (0.33-0.67) |
| 2-10 years | 114 | 18,147.7 | 6.28 | (5.23-7.55) | 0.70 | (0.48-1.01) |

*MH adjusted for maternal vital status. HR are within each age category to each baseline.
